# *Mandragora autumnalis* Distribution, Phytochemical Characteristics, and Pharmacological Bioactivities

**DOI:** 10.3390/ph18030328

**Published:** 2025-02-26

**Authors:** Ghosoon Albahri, Adnan Badran, Zaher Abdel Baki, Mohamad Alame, Akram Hijazi, Anis Daou, Joelle Edward Mesmar, Elias Baydoun

**Affiliations:** 1 Doctoral School of Science and Technology-Platform of Research and Analysis in Environmental Sciences (EDST-PRASE), Beirut P.O. Box 657314, Lebanon; ghosoon.albahri.1@ul.edu.lb (G.A.); alamefs@hotmail.com (M.A.); akram.hijazi@ul.edu.lb (A.H.); 2 Department of Biology, Faculty of Arts and Sciences, American University of Beirut, Riad El Solh, Beirut 1107, Lebanon; jm104@aub.edu.lb; 3 Department of Nutrition, University of Petra Amman Jordan, Amman P.O. Box 961343, Jordan; abadran@uop.edu.jo; 4 College of Engineering and Technology, American University of the Middle East, Egaila 54200, Kuwait; zaher.abdelbaki@aum.edu.kw; 5 Faculty of Sciences, Kut University College, Wasit 52001, Iraq; 6 Pharmaceutical Sciences Department, College of Pharmacy, QU Health, Qatar University, Doha P.O. Box 2713, Qatar

**Keywords:** *Mandragora autumnalis*, phytochemistry, antioxidant activity, antimicrobial activity, anticancer activity, antidiabetic, anti-enzymatic, toxicological activities

## Abstract

In the Mediterranean and Himalayan regions, the genus *Mandragora* (family Solanaceae), sometimes called mandrake, is widely utilized in herbal therapy and is well-known for its mythical associations. **Objective**: To compile up-to-date information on *M. autumnalis*’s therapeutic properties. Its pharmacological properties and phytochemical composition are particularly covered in managing several illnesses, including diabetes, cancer, and heart disease. **Methods:** Articles on the review topic were found by searching major scientific literature databases, such as PubMed, Scopus, ScienceDirect, SciFinder, Chemical Abstracts, and Medicinal and Aromatic Plants Abstracts. Additionally, general online searches were conducted using Google Scholar and Google. The time frame for the search included items released from 1986 to 2023. **Results:** *Mandragora* has been shown to contain a variety of phytochemicals, including coumarins, withanolides, and alkaloids. The pharmacological characteristics of *M. autumnalis*, such as increasing macrophage anti-inflammatory activity, free radicals inhibition, bacterial and fungal growth inhibition, cytotoxic anticancer activities in vivo and in vitro against cancer cell lines, and enzyme-inhibitory properties, are attributed to these phytochemicals. Furthermore, *M. autumnalis* also inhibits cholinesterase, tyrosinase, α-amylase, α-glucosidase, and free radicals. On the other hand, metabolic risk factors, including the inhibition of diabetes-causing enzymes and obesity, have been treated using dried ripe berries. **Conclusions:** Investigations into the pharmacological and phytochemical characteristics of *M. autumnalis* have revealed that this plant is a rich reservoir of new bioactive substances. This review aims to provide insight into the botanical and ecological characteristics of *Mandragora autumnalis*, including a summary of its phytochemical components and antioxidant, antimicrobial, antidiabetic, anticancer, enzyme-inhibitory properties, as well as toxicological implications, where its low cytotoxic activity against the normal VERO cell line has been shown. More research on this plant is necessary to ensure its efficacy and safety. Still, it is also necessary to understand the molecular mechanism of action behind the observed effects to clarify its therapeutic potential.

## 1. Introduction

Since prehistoric times, plants have been used extensively in medicine to treat various illnesses. Population growth, the scarcity of some treatments, their expensive cost, and the unfavorable side effects of many synthetic drugs have all contributed to a rise in commercial and scientific interest in medicinal plants [1,2]. Researchers may identify promising opportunities for medicinal development by working with indigenous tribes and examining the traditional usage of botanicals. The process of finding new drugs is streamlined when traditional knowledge and contemporary scientific methods are combined [3]. Atropine, cocaine, ephedrine, colchicine, caffeine, digitoxin, morphine, quinine, scopolamine, theobromine, and taxol are plant-derived natural products of medical importance that were previously obtained from herbal sources but are now produced economically primarily through synthesis [4]. For instance, a significant class of drugs called atropine derivatives is made from a primary constituent found in natural products [5]. This ongoing research emphasizes how important nature is as a source of bioactive molecules that support the development of novel drugs and therapies in contemporary medicine. Herbal treatments have so far been standardized using drug development techniques to find analytical marker biomolecules [6]. The innovative use of biotechnology on plants to create medications derived from natural products that the medical community can then use to treat life-threatening conditions like asthma, influenza, cancer, tuberculosis, diabetes mellitus, coronary artery disease, and diarrhea is known as plant-made pharmaceuticals [7]. However, many herbal products are still untested, and their use is either poorly or not monitored at all, even though therapies involving these agents have demonstrated promising potential and the effectiveness of many of them has been established.

**Figure 1:**
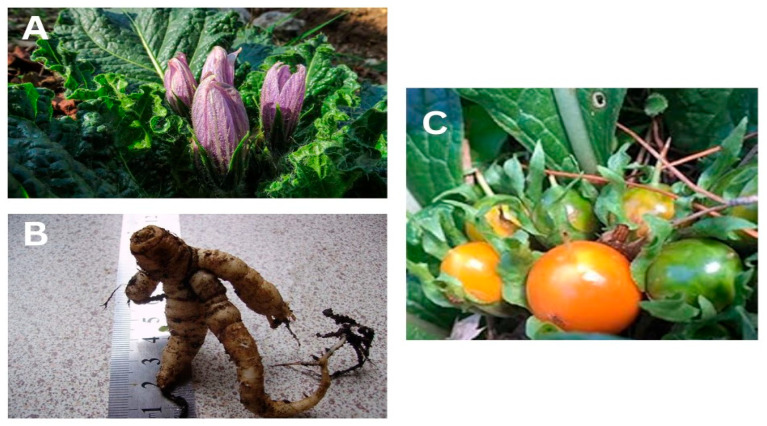
(**A**) *Mandragora autumnalis* leaves and flowers, (**B**) *Mandragora autumnalis* roots, and (**C**) *Mandragora autumnalis* fruits.

One of these botanicals is *Mandragora autumnalis*, also known as the mandrake plant or witch’s or devil’s herb, which has long been valued as one of the most important medicinal plants and as an herb with great cultural significance [8,9]. *Mandragora autumnalis* is a thick-rhizome-bearing annual herb with oblong-ovate leaves and blue-violet flowers that bloom from September through October (rarely in April) [10,11]. This plant was originally believed to have aphrodisiac and magical qualities because of the “human body”-shaped root (Figure 1) [12]. Traditionally, this species has been used to treat a wide range of conditions, including depression, melancholy, rheumatic pain, hemorrhoids, insomnia, and dysuria. Studies conducted in vitro have validated the biological characteristics of crude extracts from various parts of *Mandragora*, including their ability to modulate immunity, inhibit bacterial growth, prevent free radical-induced tissue damage, and inhibit several enzymes [13]. Indeed, *Mandragora* has a wide range of uses, including medicinal, hallucinogenic, and fertility-boosting applications, as well as the treatment of a variety of illnesses, including ulcers, inflammation, sleeplessness, and eye disorders. Specifically, *Mandragora autumnalis* has been shown to have antibacterial, antioxidant, and antitumor properties in addition to exhibiting a narcotic effect [14]. A variety of phytochemicals have been isolated from *Mandragora* species, including lipid-like compounds (β-sitosterol), coumarins (umbelliferone and scopoletin), alkaloids (atropine and scopolamine), and withanolides (salpichrolide C). These plant compounds are known to have biologically significant attributes, such as anticholinergic, antidepressant, antioxidant, and anti-inflammatory properties [15]. Most published publications related to *Mandragora autumnalis* concentrate on the unique ecological role of the mandrake plant despite the apparent increase in interest in its pharmaceutical effects. The discovery of novel bioactive metabolites with potential medical applications is necessary due to the ongoing need for alternative treatments derived from *Mandragora* species. This review attempts to gather all the previous research and provide a foundation for further studies to help identify *M. autumnalis*’s possible use as a medicinal agent. Considering the paucity of research on the phytochemical makeup and pharmacological characteristics of *Mandragora autumnalis*, this review paper aims to describe the plant’s botanical and ecological traits, provide an updated, thorough compilation of its phytochemical composition, and discuss its pharmacological activities and toxicological effects. Our review primarily concentrates on gathering all available information, both current and historical, regarding the pharmacological characteristics of this species, laying the groundwork for further research on this important species in the medical domain.

## 2. Methods

**Figure 2:**
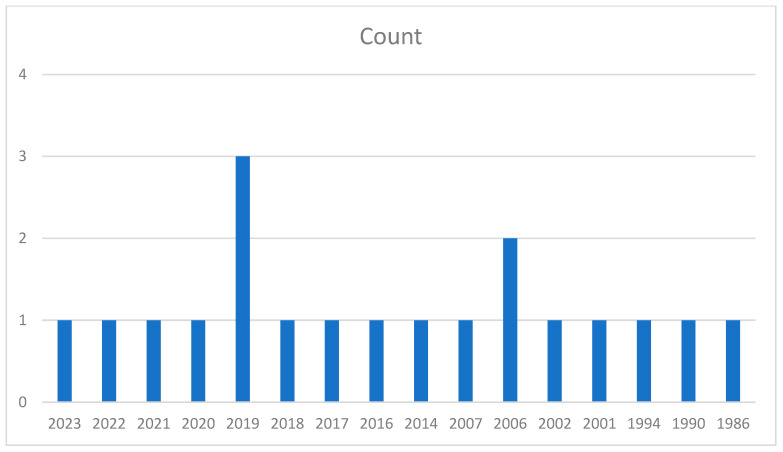
A bar graph showing the number of articles considering *Mandragora autumnalis* from the year 1986 to 2023.

Articles on the review topic were found and retrieved using major scientific literature databases, such as PubMed, ScienceDirect, Scopus, Chemical Abstracts, Henriette’s Herbal Homepage, and Medicinal and Aromatic Plants Abstracts. The general internet Google Scholar and Google were also used for searches. Based on PubMed, Figure 2 shows that the search period encompassed the number of publications released from 1986 to 2023. The keywords used are “Mandrake plant”, “*Mandragora* species”, “*Mandragora autumnalis*”, “phytochemistry”, “extraction”, “ethnopharmacology”, “antioxidant activities”, “antibacterial”, “anti-enzymatic”, “anticancer activities”, “clinical trials”, and “toxicological activities”.

## 3. Taxonomical Classification of *Mandragora autumnalis*

The *Mandragora* genus belongs to the family Solanaceae, which has six species that are native to the Eurasian continent, including *M. turcomanica*, *M. officinarum*, *M. caulescens*, *M. autumnalis*, *M. acaulis*, and *M. vernalis* [12]. The taxonomy of *Mandragora autumnalis* is shown in Table 1.

## 4. Geographical and Botanical Characteristics of *Mandragora* L.

### 4.1. Geographical Characteristics

**Figure 3:**
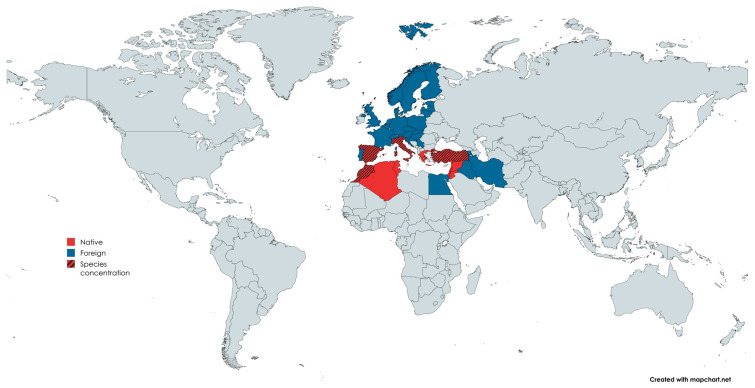
Geographic distribution of native and grown (foreign) *Mandragora* L.; the lined legend corresponds to the regions where *Mandragora autumnalis* species are concentrated. The map was designed using https://www.mapchart.net/world.html. Accessed online on 25 February 2025.

*Mandragora* L. is found throughout northern Africa and Eurasia. The genus has a traditional disjunction pattern in the biogeography of the Old-World flora, occurring discontinuously in the Tibetan Plateau, the Mediterranean area, and the Turanian region [16]. This plant is native to the following regions: Turkey, Palestine (Israel), Jordan, Syria, Lebanon, Tunisia, Algeria, and Morocco; on the other side, the plant is not native and is not ubiquitous according to the Flora Croatica Database, 2022, to Western, Central, and Northern Europe, Iran, Iraq, Serbia, Croatia, Armenia, and Egypt [17], as shown in the map in Figure 3.

### 4.2. Botanical Characteristics of Mandragora autumnalis

The wild medicinal plant *Mandragora autumnalis* is a perennial herbaceous plant belonging to the Solanaceae family [8]. The specific name *autumnalis* refers to the season in which these plants typically bloom. Mandrake has a ground-hugging rosette of large shiny green leaves up to 40 cm long and densely packed mauve or violet five-petalled flowers that are 4 to 5 cm across [18]. Its ripe fruits (berries) are orange or yellow, and its blossoms are purple or violet. Unripe fruits, leaves, and roots are known locally as Tufah Almajan (Satan’s Apple), love apples, and Beed Aljin (Eggs of the Jinn) because of their propensity to stimulate the senses and produce mental disorders [19]. There are 55 main odoriferous components in the relatively strange chemical makeup of mandrake scent [20].

## 5. Phytochemical Constituents of *Mandragora autumnalis*

In addition to its ecological value, the mandrake plant has been utilized in traditional folk medicine to cure several illnesses, such as skin issues, inflammatory symptoms, and digestive difficulties. Additionally, it has been demonstrated that extracts from *Mandragora autumnalis* exhibit various pharmacological properties, including antibacterial, anti-fungal, anti-enzymatic, and anticancer properties. Throughout the years, different parts of the mandrake plant have been used to treat various ailments, as shown in Table 2. Numerous secondary metabolites from other parts of *M. autumnalis* are thought to be responsible for these characteristics.

Tropane alkaloids found in abundance in *M. autumnalis* roots include scopolamine, and L-hyoscyamine, which, when extracted, yield the racemic combination of L- and R-hyosciamine, often referred to as atropine [27]. Additional tropane alkaloids derived from the roots include calystegines, α- and β-belladonnine, apoatropine, 3α-tigloyloxytropane, and 3,6-ditigloyloxytropane. Cuscohygrine, an alkaloid of pyrrolidine, was also produced [26], and belladonnines were absent from the fresh roots but present in the dried roots [28]. Moreover, an earlier study showed that the ethanol extract of *M. autumnalis* leaves had significant levels of flavonoids and phenols along with other phytochemicals such as terpenoids, anthraquinones, coumarins, phlobatannins, and tannins [13]. According to LC-MS analysis of the ethanol crude extract of *M. autumnalis* leaves, quercetin 3,4′-diglucoside was detected in large proportions, followed by quinic acid, chlorogenic acid, and quercetin 4′-O-glucoside [29]. Four flavonoids were extracted from the mature fruit of *M. autumnalis* using ethyl acetate extract, and by comparing their 1H- and 13C-NMR spectrum analyses with those published in the literature, their structures were determined to be kaempferol, luteolin, myricetin, and taxifolin [13]. Furthermore, it was discovered that the main constituents of *M. autumnalis* berries were ethyl esters of middle-chain acids, along with ethyl caprylate, linoleic acid, n-hexyl acetate, ethyl caprate, and ethyl caproate [30]. Table 3 lists the most important phytochemical composition of *M. autumnalis* roots, leaves, fruits, and essential oils extracted from *M. autumnalis* seeds, including the extraction solvent and the methods used to identify these compounds.

**Figure 4:**
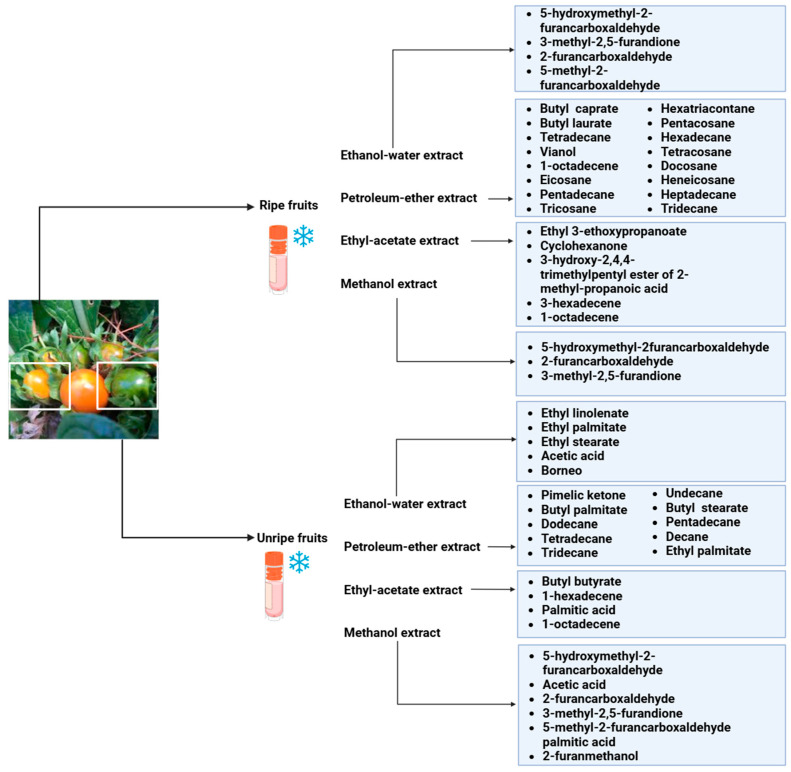
The major compounds isolated from lyophilized ripe and unripe fruits of *Mandragora autumnalis* using different extraction solvents (the figure was designed using https://app.biorender.com/). Accessed online on 25 February 2025.

A total of 23 n-alkanes, 1 branched-chain alkane, 2 cyclohexanes, 8 alkenes, 2 branched-chain alkenes, 3 alcohols, 3 aldehydes, 6 ketones, 8 heterocyclic compounds, 4 thio compounds, 6 benzene hydrocarbons, 3 phenols, 18 carboxylic acids, and 48 carboxylic acid esters were among the 125 compounds identified in the fruit extracts of *Mandragora autumnalis* after the seeds were removed. The major compounds of different extracts of ripe and unripe *M. autumnalis* fruit are shown in Figure 4 [30,32,33,34]. Many identified compounds contribute to the fruit’s pleasant aroma and taste, albeit in different amounts, where ripe fruit contains significant “odor compounds”. Solavetivone is a representative phytoalexin that has been isolated from potato (*Solanum tuberosum*) tubers infected with the blight fungus *Phytophthora infestans* [35], air-cured tobacco (*Nicotiana tabaccum*) leaves [36], and several Solanaceae species. Furthermore, furan derivatives, especially 5-hydroxymethyl-2-furancarboxaldehyde (HMF), were present in higher amounts in methanolic extracts of ripe fruits (81.4%) than in unripe (57.0%) fruits, which may be explained by the fact that the higher HMF concentration in ripe fruits was proportionate to their higher sugar concentration. However, according to research by Muratore et al. on heated grapes, the HMF concentration was also highly affected by the change in temperature, pH, and sugar concentration; however, the HMF levels were higher in the methanolic extracts of unripe fruit than in those of ripe fruit, suggesting that methanol interacted in a sugar-independent manner [23]. *Mandragora autumnalis* compositional studies are insufficient, as the previously cited investigations demonstrated. Thus, a thorough examination of the phenolic compounds and flavonoids present in *M. autumnalis* extracts, especially the leaves and roots, as well as other classes of bioactive metabolites, should be the main goal of future research.

## 6. Pharmacological Bioactivities of *Mandragora autumnalis*

### 6.1. Antioxidant Activities

Reactive oxygen species (ROS) production and accumulation are out of balance with the body’s capacity to detoxify through antioxidant defenses [37]. This imbalance leads to oxidative stress, which causes premature aging and increases the risk of some diseases, including cancer, inflammatory disorders, diabetes, and neurodegenerative diseases [38,39]. Growing interest has been shown in the function of antioxidants in preventing oxidative damage in recent years [40]. Synthetic antioxidants have been widely used to lessen oxidative stress. These compounds are frequently used as food and consumer product preservatives and have been linked to dubious toxic and cancer-causing effects. Food-produced peroxides can interact with synthetic phenolic antioxidants, and research has demonstrated their harmful and cancer-causing impacts [41]. Propyl gallate (PG), propylated hydroxyanisole (BHA), propylated hydroxytoluene (BHT), and tertiary butylhydroquinone (TBHQ) are the most common synthetic phenol derivatives [42,43]. Yet, considering issues about these antioxidants’ safety, natural sources like plants have been used instead because they contain bioactive metabolites with potent antioxidant activity [44]. The antioxidant activities of various *M. autumnalis* extracts are displayed in Table 4.

The strong scavenging actions of the *M. autumnalis* flavonoid fruit fraction against the 1,1-diphenyl-2-picrylhydrazyl (DPPH) assay demonstrated the presence of antioxidants. The extract had an IC_50_ value of 5.37 ± 0.41 µg/mL, which is comparable to the standard antioxidant Trolox (IC_50_ = 2.23 ± 1.23 µg/mL) in terms of free radical scavenging properties [13]. Moreover, other research studied the antioxidant activity of the methanolic extract of *M. autumnalis* leaves (L-Met) and flowers (F-Met), as well as the acetone extract of the leaves (L-Ac) and flowers (F-Ac) using several assays. The phosphomolybdenum method was used to assess the total antioxidant capacity [47]. The extracts’ overall antioxidant capacity was greater in L-Ac (1.98 mmol TE/g extract). However, when using a DPPH assay [48] and compared to the other extracts, F-Met has the strongest free radical scavenging activity, with 73.09 mg TE/g of extract. In terms of metal-chelating activity [49], F-Met had the highest level (15.94 mg EDTA/g). Furthermore, The FRAP and CUPRAC assays are frequently used to assess the plant’s antioxidant capacity [50,51]; as a result, the methanolic extract of the flowers showed the highest antioxidant activity based on these two methods. Additionally, previous research assessed the DPPH radical scavenging activity of the aqueous root extract and synthesized silver nanoparticles of *M. autumnalis*; both extracts showed significant dose-dependent antioxidant activities, with an IC_50_ of 47.16 ± 0.41 and 51.81 ± 0.10 µg/mL [46]. Thus, *M. autumnalis* has strong antioxidant properties that demand more research. Green extraction techniques that produce higher concentrations of antioxidant compounds from plants have recently attracted more attention to promote longevity, reduce aging effects, and prevent disorders linked to oxidative stress. It should be noted that in vitro assays, which are frequently prone to errors because of the chemical diversity of phytochemicals, have been used to evaluate *M. autumnalis*’ antioxidant capacity, which should be carefully interpreted and backed up in future studies that concentrate on using in vivo techniques to measure antioxidant activity.

### 6.2. Antimicrobial Activities

The widespread and inappropriate use of antibiotics has resulted in antimicrobial resistance, representing a major global challenge and serious threat to human health [52]. Due to the declining effectiveness of traditional drug therapies, there has been a pressing need to develop new antimicrobial agents using novel therapies based on natural metabolites because of their chemical diversity and efficacy [53]; in fact, extracts from medicinal plants have been widely reported to exhibit antimicrobial activities, acting by both increasing the activity of antibiotics and inhibiting the growth of various pathogens, including bacteria, fungi, and viruses [54,55]. This has made it necessary to look for and create new antimicrobial agents using natural compounds. Because medicinal plants produce bioactive compounds with known therapeutic properties, they have been thoroughly investigated as possible antimicrobial agents. Several studies have assessed the antibacterial efficacy of *M. autumnalis* extracts, as shown in Table 5.

A microdilution assay was employed to evaluate the antimicrobial activity of the fruit flavonoid fraction of *M. autumnalis*. The findings demonstrated that this fraction possesses the strongest antibacterial activity against the *K. pneumoniae* strain, higher than ampicillin and comparable to the reference medication ciprofloxacin. In addition, this fraction showed antibacterial inhibitory effects against *S. aureus*, *E. coli*, *P. aeruginosa,* and Methicillin-resistant *Staphylococcus aureus* (MRSA) and strong antifungal activity against *C. albicans* when compared with the commercial antifungal drug fluconazole [13]. Moreover, it was discovered that *P. aeruginosa* bacteria are more sensitive to silver nanoparticles synthesized from *M. autumunalis* aqueous roots extract (Ma-AgNPs) compared to *M. autumnalis* roots extract, which showed the highest sensitivity against *B. subtilis* [46]. The MIC values for *M. autumnalis* ethanol leaf extract and fractions were ascertained using a microdilution assay. Gram-positive bacteria (*B. subtilis*) and Gram-negative bacteria (*P. aurignosa*) were vulnerable to the antibacterial properties of ethanol extract and n-hexane fraction. Additionally, the n-hexane fraction demonstrated antifungal activity against *Candida albicans*, with a minimum inhibitory concentration (MIC) of 12.5 mg/mL. However, in both bacterial and fungal tests, aqueous/methanol and aqueous fractions exhibited no activity [29]. These studies support the potential use of *M. autumnalis* extracts as antibacterial agents. However, investigating the mechanism underlying the observed antibacterial activity is necessary.

Further research should determine how *M. autumnalis* extracts work against other pathogens. Together, these findings suggest that *M. autumnalis* may provide new antimicrobial agents that could aid in botanical screening efforts to find new medications to combat antibiotic resistance and guarantee future antibiotic-free management of microbial growth.

### 6.3. Anticancer Activities

**Figure 5:**
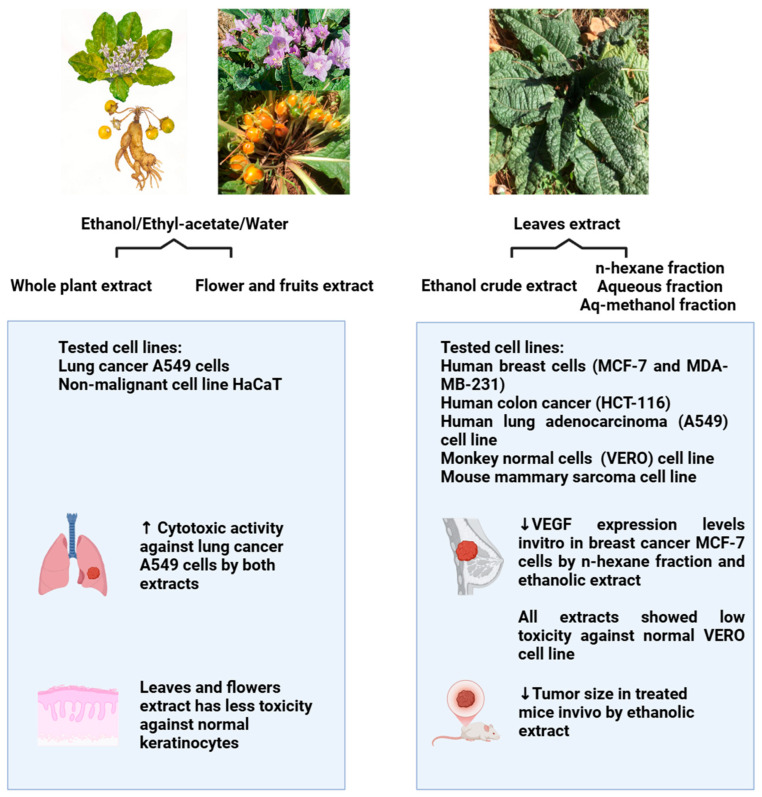
The anticancer activities of *M. autumnalis* against various cancer cell lines (the figure was designed using https://app.biorender.com/). Accessed online in 25 February 2025.

Cancer remains a major cause of death globally, even with significant progress in cancer treatment [56,57]. Globally, cancer is the leading cause of death and a serious public health concern [58]. In addition, traditional cancer treatment plans are frequently linked to negative side effects and multidrug resistance, which has raised interest in the hunt for novel bioactive compounds derived from plants [59,60]. In this context, several investigations have evaluated *M. autumnalis*’s anticancer capabilities. The anticancer activity of extracts of *Mandagora autumnalis*’s flowers, fruits, and whole plant (extracted using equal proportions of ethanol/ethyl acetate/water) was shown to be 2.7- and 3.5-fold more cytotoxic to (A549) lung cancer cells than to non-cancerous human keratinocyte (HaCat) cells, respectively [61]. Moreover, another study evaluated the anticancer activity of *Mandragora autumnalis* crude ethanolic leaf extract and several leaf fractions (n-hexane, aqueous, and aqueous-methanolic) [14]. To assess the anticancer effect of *M. autumnalis* leaves in both in vitro and in vivo models, ethanol crude extract and solvent fractions were extracted. By using the 3-(4,5-Dimethylthiazol-2-yl)-2,5-Diphenyltetrazolium Bromide (MTT) method, five cancer cell lines—human lung adenocarcinoma (A549), human colon cancer (HCT-116), human breast cancer (MCF-7 and MDA-MB-231), a mouse mammary sarcoma cell line (EMT6/p), and one normal monkey cell line (VERO)—were evaluated in vitro [62,63,64,65,66,67]. Low cytotoxicity against the normal VERO cell line, the downregulation of VEGF expression, and the strongest antitumor activity against MCF-7 breast cancer cells were demonstrated via ethanol extract and n-hexane fractions. Furthermore, mice administered *M. autumnalis* ethanol extract showed a significant reduction in tumor size when compared to the negative control [14]. To assess the anticancer potential of various *M. autumnalis* extracts, the referenced studies employed cell viability assays; these investigations demonstrated the potent anticancer properties of the plant’s extracts and the isolated compounds mentioned above against a variety of cancer types and cancer hallmarks. Figure 5 depicts *M. autumnalis*’s intriguing anticancer properties; Table 6 summarizes these properties. However, further research must confirm *M. autumnalis*’s anticancer potential and examine the underlying molecular mechanisms.

### 6.4. Antidiabetic, Anti-Enzymatic, and Anti-Obesity Activities

Research suggests that *Mandragora autumnalis* may have antidiabetic effects. Studies have shown that extracts from *M. autumnalis* can inhibit enzymes like α-amylase and α-glucosidase [68]. These enzymes play a crucial role in the digestion of carbohydrates, and inhibiting them can help slow down the absorption of glucose into the bloodstream [69]. This can be beneficial for managing blood sugar levels in people with diabetes hyperglycemia or elevated blood glucose concentration, which is a hallmark of diabetes, a chronic metabolic disease caused by an insufficient or malfunctioning insulin supply [70]. Serious complications in many organs, such as kidney and vision loss, heart attacks, and strokes, are caused by uncontrolled diabetes [71,72]. On the other hand, the majority of patients with type 2 diabetes have dyslipidemia, which is characterized by high triglycerides, low HDL-C, and a preponderance of small-dense LDL particles. Between 60% and 70% of diabetic patients have at least one lipid abnormality, though not all patients show all symptoms [73]. Previous research showed that the majority of patients with elevated lipase also have elevated amylase [74], and since herbal medicine is inexpensive and has few adverse effects, it has been preferred over synthetic medications for the treatment of diabetes. Insulin plays a crucial role in preserving glucose homeostasis by increasing GLUT4 translocation to the muscle’s plasma membrane (PM) [75]. When muscle cells were treated with flavonoid fruit fraction both with and without insulin, a dose-dependent increase in GLUT4 translocation was observed. When no insulin treatment was administered, the fruit flavonoid fraction at concentrations of 0.125, 0.25, and 0.5 µg/mL significantly increased GLUT4 translocation by 1.33 ± 0.13, 1.33 ± 0.22, and 1.56 ± 0.21 µg/mL, respectively. Additionally, the fraction significantly increased GLUT4 translocation to the PM in the presence of insulin by 2.35 ± 0.43 and 2.35 ± 0.19 at 0.25 and 0.5 mg/mL, respectively [13]. Additionally, the results showed that the fraction exhibited dose-dependent α-amylase and α-glucosidase inhibitory activity in comparison to the commercial antidiabetic therapeutic agent acarbose. Furthermore, the fraction exhibited dose-dependent pancreatic lipase inhibitory activity in comparison to orlistat [13]. Moreover, other research showed that the acetone extract exhibited greater activity for α-amylase compared to methanol extract. According to the α-amylase assay, the leaves and flower acetone extracts correspond to the highest amylase inhibitory activity compared to the methanolic leaves and flower methanolic extracts. However, only the acetone extract of the leaves extract did not exhibit any α-glucosidase inhibitory activity, whereas the rest of the extracts exhibited mild α-glucosidase inhibitory activity. All extracts were found to have stronger inhibitory effects against α-amylase than α-glucosidase [45]. Table 7 illustrates the anti-enzymatic bioactivities of *Mandragora autumnalis*. Furthermore, there are serious and occasionally lethal side effects associated with some synthetic antidiabetic drugs. Therefore, over the past 20 years, scientists have concentrated their research on natural products like flavonoids as possible alternatives to acarbose and other antidiabetic drugs.

### 6.5. Anticholinesterase and Anti-Tyrosinase Activities

Alzheimer’s disease is the most common cause of dementia and is a neurodegenerative disorder of the brain that is clinically characterized by a progressive decline in cognitive abilities [76]. Acetylcholine deficiency or loss is linked to a marked decrease in central cholinergic neurotransmission [77]. The primary treatment approach is to restore acetylcholine levels by inhibiting the enzymes butyrylcholinesterase (BChE) and acetylcholinesterase (AChE), which hydrolyze acetylcholine [78,79]. On the other hand, the term “Parkinson’s disease dementia” (PDD) refers to the growing recognition of dementia in Parkinson’s disease (PD) cases [80]. The strongest neuropathological correlate of developing dementia in Parkinson’s disease appears to be the spread of fibrillar α-synuclein (α-syn) pathology from the brainstem to limbic and neocortical structures; novel treatment approaches involve blocking brain tyrosinase levels and neuromelanin production [81]. Thus, previous research that studied the inhibition activity *M. autumnalis*’s cholinesterase inhibitory properties was evaluated concerning the Alzheimer’s disease-related enzymes AChE and BChE. The results showed that the acetone extract of the leaves demonstrated notable inhibitory activity on BChE, whereas the methanolic extract of the flower extract had the strongest activity on AChE. Moreover, the tyrosinase inhibitory activity of the acetone extract of the leaves was the highest, at 29.68 mgKAE/g extract, whereas the methanol extract of the flower showed no tyrosinase-inhibiting properties. The hydroxyl group of phenolic compounds in *M. autumnalis* may be responsible for inhibiting tyrosinase activity [82]. These findings pave the way for the future usage of *M. autumnalis* for treating or decreasing the symptoms of Alzheimer’s and Parkinson’s disease. The anti-enzymetic activities of different extracts of *Mandragora autumnalis* are summarized in Table 8.

### 6.6. Anti-Inflammatory Activities

**Figure 6:**
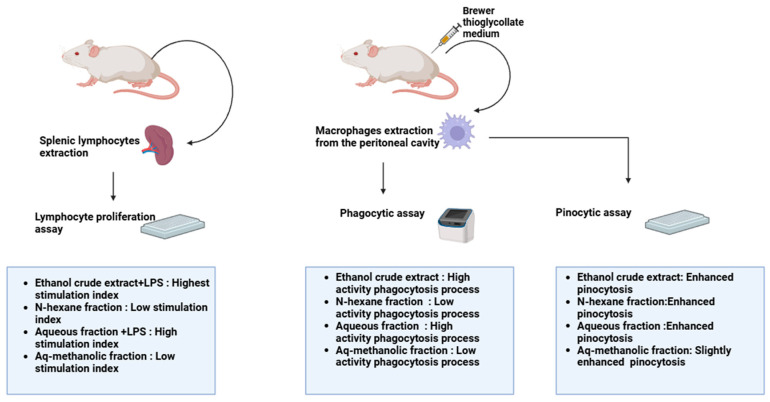
The immunomodulatory effects of different extracts of *M. autumnalis* leaves (the figure was designed using https://app.biorender.com/). Accessed online on 25 February 2025.

The body uses inflammation as a self-defense and homeostatic mechanism, always maintained under homeostatic control. On the other hand, chronic or excessive inflammation can lead to the development of several illnesses, including cancer, diabetes, and cardiovascular disease [38,83]. An important component of the acquired immune system, lymphocytes’ capacity to proliferate is thought to be a measure of the degree of cell immunity [84]. In the presence and absence of mitogens, including lipopolysaccharide (LPS), which stimulates B cells, and Concanavalin A (con A), which stimulates T cells, lymphocyte proliferation was induced, and the outcomes were roughly comparable. The aqueous fraction and the ethanol extract containing lipopolysaccharide (LPS) had the highest stimulation index values: 2.5 and 3.6, respectively. Conversely, the aqueous/methanol fractions and n-hexane demonstrated reduced activity at the highest concentration, with low stimulation index values [13]. Macrophage activity was increased by ethanol extract and the aqueous fraction of *M. autumnalis* leaves through phagocytosis. However, the n-hexane and aqueous/methanol fractions, which had phagocytic indices of 143 and 165, respectively, demonstrated less activity. Furthermore, the ethanol crude extract, aqueous, and n-hexane fractions enhanced the macrophages’ capacity for pinocytosis [13]. The immunomodulatory effects of different extracts of *M. autumnalis* leaves are summarized in Table 8 and illustrated in Figure 6.

**Table 8:** The anti-inflammatory effects of different extracts of *M. autumnalis* leaves.

Extract	Dose	Experimental Model	Observation	References
Ethanolic crude extract	4 mg/mL	-Lymphocyte proliferation assay -Phagocytic assay -Pinocytosis assay	-Highest activity, with an LPS index value of 3.6 -Highest phagocytotic index of 325 -Highest pinocytotic index of 208	[13]
n-hexane fraction	4 mg/mL	-Lymphocyte proliferation assay -Phagocytic assay -Pinocytosis assay	-Lowest activity with Con A -Least phagocytotic index of 143 -Highest pinocytotic index of 208	[13]
Aqueous fraction	4 mg/mL	-Lymphocyte proliferation assay -Phagocytic assay -Pinocytosis assay	-Highest activity with LPS, with an index value of 2.5 -High phagocytotic index of 298 -High pinocytotic index of 200	[13]
Aqueous-methanolic fraction	4 mg/mL	-Lymphocyte proliferation assay -Phagocytic assay -Pinocytosis assay	-Lowest activity with Con A -Low phagocytotic index of 165 -High pinocytotic index of 200	[13]

### 6.7. Green Nanotechnology

**Figure 7:**
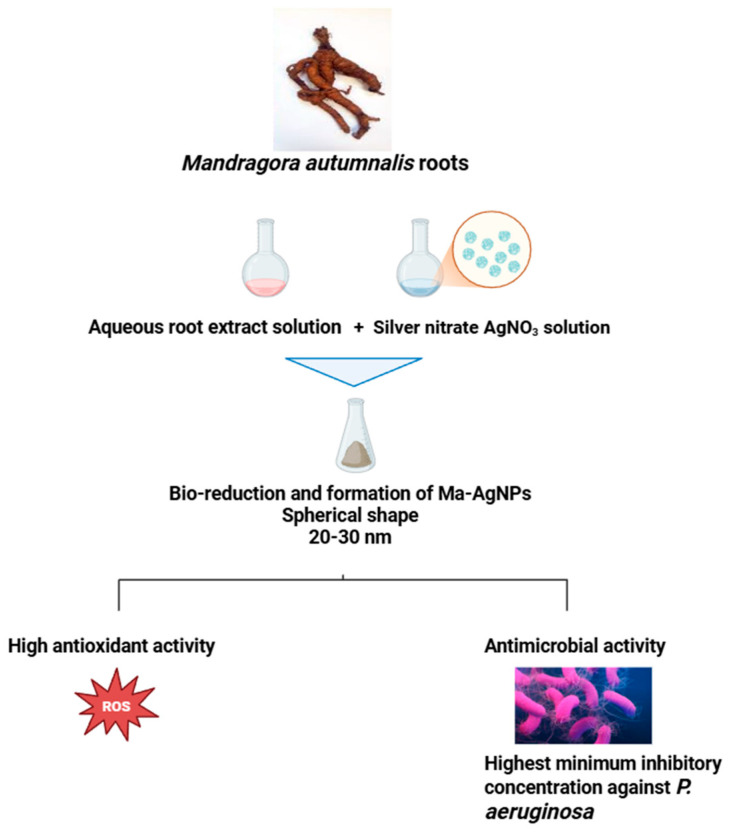
*Mandragora autumnalis* roots produce silver nanoparticles (AgNPs) with antioxidant and antibacterial properties (the figure was designed using https://app.biorender.com/). Accessed online on 25 February 2025.

Nanotechnology is a pioneering field that has profound effects on consumer goods and is transforming several industries [85,86]. This rapidly growing field of study (nanoscience and nanotechnology) deals with systems, devices, and structures with unique characteristics and capabilities because of the way their atoms are arranged on the 1–100 nm scale [87]. Generations of nanomaterials have surfaced and are employed in interdisciplinary scientific domains; nanoscale manufacturing will soon be integrated into practically every field of science and technology, as evidenced by the quick development of nanoscience [88,89]. Silver nanoparticles (AgNPs) are tiny silver particles that are produced chemically or environmentally [90]. They are affordable and possess special physicochemical qualities. Silver nanoparticles are useful in a variety of applications, including textiles, biomedical applications, protective surface coatings, and wound healing, due to their well-known antimicrobial and antiviral properties [91]. AgNPs can be produced conventionally using physical techniques, like laser ablation, chemical processes, like reduction reactions, and green synthesis routes using biomaterials like fungi, bacteria, and plant extracts. The green synthesis of AgNPs guarantees the creation of environmentally benign and nontoxic nanoparticles with improved stability, biocompatibility, and biological activity [92,93,94,95]. The potential of *M. autumnalis*-mediated AgNPs for antioxidant activity was examined using an in vitro DPPH test system to assess the antioxidant activities of nanoparticles synthesized from *M. autamnalis* aqueous root extract (Ma-AgNPs). Ma-AgNPs were found to be potent free radical scavengers, demonstrating effective inhibitory activity in a dose-dependent fashion [46]. Moreover, the antibacterial properties of Ma-AgNPs were examined in Gram-negative (*Escherichia coli* and *Pseudomonas aeruginosa*) and Gram-positive (*Bacillus subtilis* and *Staphylococcus aureus*) bacteria. The results demonstrated that Ma-AgNPs exhibit antibacterial activity against every tested bacterial strain when present at a concentration of 25 µg/mL, and it was discovered that *P. aeruginosa* bacteria were more sensitive to Ma-AgNPs than any other bacteria tested [46]. The antibacterial and antioxidant properties of AgNPs produced by *M. autumnalis* are depicted in Figure 7. When taken together, these findings highlight the potential of *M. autumnalis* in environmentally friendly nanoparticle synthesis and call for more research in this field.

### 6.8. Toxicological Studies

There are growing concerns about the safety profile of herbal medicinal products as their use spreads throughout the world. It is not required to assess these products’ safety or toxicity before going on sale because most of them are categorized as foods or dietary supplements in most countries [2,96,97]. However, toxicological research is crucial when creating herbal medicine to guarantee efficacy, quality, and safety to prevent any potential negative health effects [98,99]. Numerous studies assessed *Mandragora autumnalis*’s safety and toxicological profile. For that reason, previous research studied the anesthetic properties of *Mandragora autumnalis* roots [100], where the commercial value of fish residues is reduced when synthetic anesthetics are used [101]. The study aimed to use *Mandragora autumnalis* roots rather than synthetic anesthetics that leave harmful residues. In vivo investigations on fish were conducted to examine mandrake root extracts and determine the proper experimental dosage. The *Mandragora* aqueous root extract obtained using a maceration method showed the best potential to be used as a fish anesthetic drug [100]. This can be related to the presence of tropane alkaloids that exhibit an anticholinergic effect by binding to either muscarinic receptors (mAChR) or nicotinic acetylcholine receptors (nAChR), which blocks the action of the neurotransmitter acetylcholine (ACh) in the central and peripheral nervous systems (CNS and PNS) [102]. This anticholinergic action affects both CNS and PNS, and symptoms include respiratory and heart rate changes, muscle contraction, hallucinations, and local anesthesia [103].

Moreover, topical exposure to *M.autumnalis* was believed to result in irritant contact dermatitis. According to reports, applying *Mandragora* root extract topically caused erythema, swelling, and burning. Erythematous, oedematous, and scaly plaque lesions were among the symptoms linked to allergic contact dermatitis brought on by *Mandragora* root sap [104,105]. Furthermore, recent research assessed the in vivo toxicity of the *Mandragora autumnalis* ethanol leaf extra by measuring liver and kidney parameters. Serum levels of aspartate aminotransferase, alanine transaminase, and creatinine were normal in the tumor-bearing mice given the *M. autumnalis* extract [14]. In general, additional toxicological analysis is required to confirm *M. autumnalis*’s safety profile for medical use.

## 7. Conclusions and Future Perspectives

Botanicals have intrigued humans since antiquity due to their extraordinary nutritional, cosmetic, and therapeutic properties. An increasing amount of information demonstrates that herbal medications have fewer adverse effects than most synthetic therapies, making them an essential source for creating novel pharmaceuticals, cosmetics, and nutritional supplements. Recently, there has been an increase in interest in research on plant products because plants are now, more than ever, considered as potential leading metabolite producers for drug development. However, plant metabolites should be optimized for efficacy and thoroughly evaluated for toxicity in plant-derived drug discovery. *M. autumnalis* has been shown in phytochemical and pharmacological studies to be a rich source of bioactive metabolites with a wide range of applications, further supporting its ethnopharmacological uses. Previous research showed that *M. autumnalis* has free radical scavenging activity, with an ability to inhibit the growth of tested microbial strains, decrease the viability of several cancer cell lines by increasing the cytotoxicity against various cancer cell lines in vitro, and decrease tumor size in experimental mice in vivo. Moreover, it increases the immunomodulatory activity of macrophages by increasing phagocytotic and pinocytotic activity, as well as increasing the proliferation potential of lymphocytes. Furthermore, it has a promising potential against diabetes and obesity. However, the identification of the bioactive metabolites mediating *M. autumnalis*’s biological activities should be another goal of future research, where drugs that target a range of illnesses could be developed using molecular mechanisms of action. The food and nutraceutical industries may also make use of these bioactive metabolites. Additionally, before being tested in clinical trials and utilized for drug development, the safety profile and effectiveness of the bioactive metabolites should be assessed using in vivo models following their identification and isolation. It is crucial to remember that the literature does not provide enough proof of *M. autumnalis*’s anticancer and anti-inflammatory properties. Toxicological studies should be conducted alongside additional research on the pharmacodynamic, pharmacokinetic, and mechanism of action of *M. autumnalis* extract to develop safe and natural pharmaceutical medications from this medicinal plant. Methods to lessen toxicity while preserving bioactivity and enhancing therapeutic effect are essential for advancing such medications to drug development and clinical trials. The elucidation of new methods should be retrieved to increase the delivery of the bioactive components of *Mandragora* and lessen its toxicological profile; for example, applying new fractionation systems to the different parts of *Mandragora autumnalis,* which will allow for the usage of effective beneficial components from this plant and eliminate the toxic related compounds. Thus, more pharmacological research is required to support the clinical efficacy of treatments, including in vitro and in vivo tests. There is currently insufficient data from clinical and even in vivo investigations to substantiate the in vitro findings. The lack of diversity in pharmacological research on *Mandragora* species can be attributed to issues with plant accessibility and toxicity.

## Figures and Tables

**Table 1:** Taxonomic classification of *Mandragora autumnalis*.

Kingdom	*Plantae*
Phylum	Tracheophyta
Class	Magnoliopsida
Order	Solanales
Family	Solanaceae
Genus	*Mandragora* L.
Species	*Mandragora autumnalis* Bertol.

**Table 2:** The traditional medical and modes of usage of different parts of *M. autumnalis*.

Plant Part	Mode of Usage	Traditional Use	Country	Reference
Roots	Warm pad	Treat tendons	Persia	[21]
Leaves	Poultice	Treat wounds	Cyprus	[22]
Roots	Soaked in vinegar	Gout	Persia	[23]
Seeds	Clyster	Uterus cleaning	Turkey North Africa	[24]
Leaves	External application	Abscesses Gland’s swellings small tumors	Egypt	[24]
Roots	Oral	Headache Snake bites Anesthesia Sedation Inflammation	Mediterranean area Europe	[25]
Leaves	Rubbed to skin	Freckles removal	Not identified	[26]
Leaves	Oral	Coughs Asthma Bronchitis	Jorden Morocco Northern Africa	[26]

**Table 3:** The most important bioactive metabolites from the roots, leaves, fruits, and essential oils extracted from *M. autumnalis,* their extraction solvent, and identification methods (all the structures were retrieved from ChemDraw Pro 8 software).

Plant Part Extract	Extraction Solvent	Compounds	Major Metabolites	Reference
Fruits	Ethyl acetate	Flavonoids	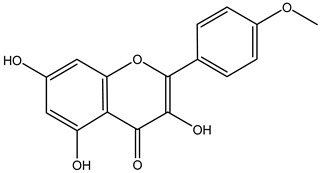 Kaempferol	[12]
			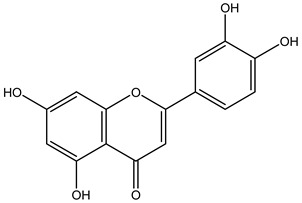 Luteolin	
			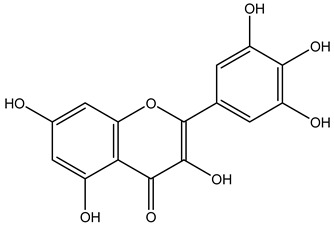 Myricetin	
			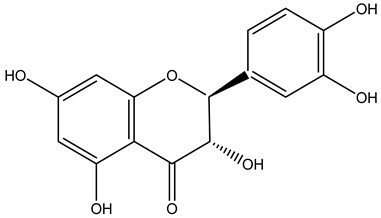 Taxifolin	
Fruits	Water distillation	Esters	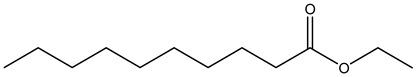 Ethyl caprate	[30,31]
			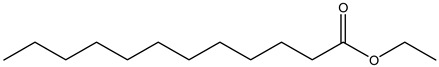 Ethyl laurate	
			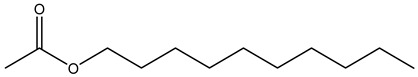 Decyl acetate	
			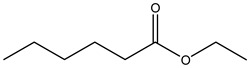 Ethyl caproate	
Leaves	Ethanolic extract	Phenolic acids	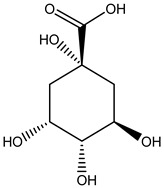 Quinic acid	[29]
			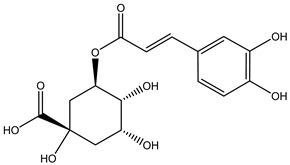 Chlorogenic acid	
			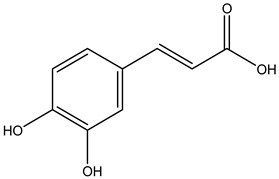 Caffeic acid	
			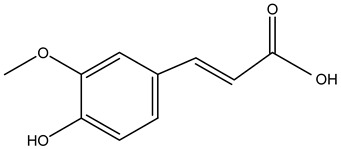 Ferulic acid	
			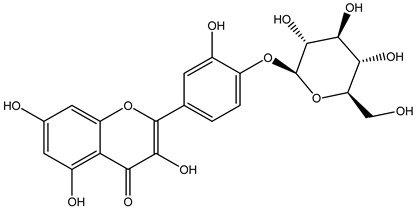 Spiraeoside	
Leaves	Ethanolic extract	Fatty acids	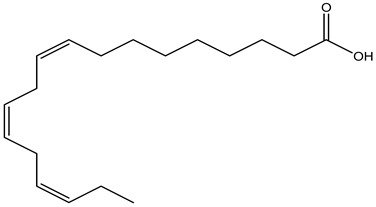 α-Linolenic acid	[14]
			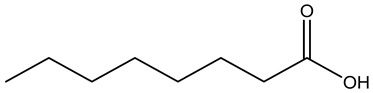 Caprylic acid	
			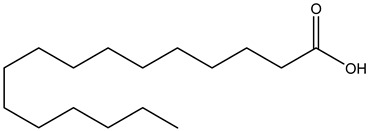 Palmitic acid	
		Esters	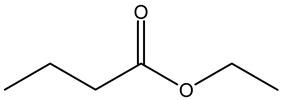 Ethyl butanoate	
		Alkanes	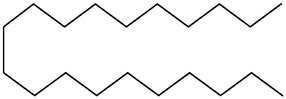 Eicosane	
		Monoterpenoids	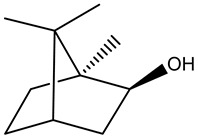 Borneol	
		Triterpenoids	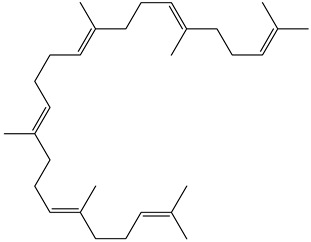 Squalene	
Roots	Methanolic extract	Alkaloids	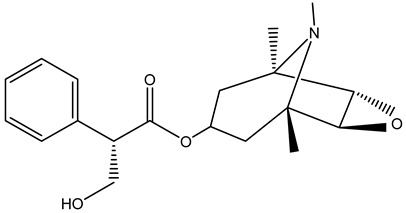 Scopolamine	[26,28]
			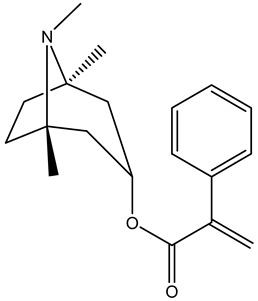 Apoatropine	
			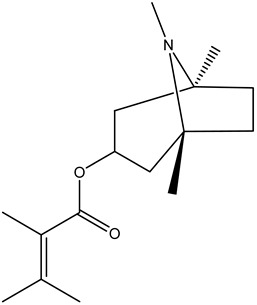 3 α-tigloyloxytropane	
			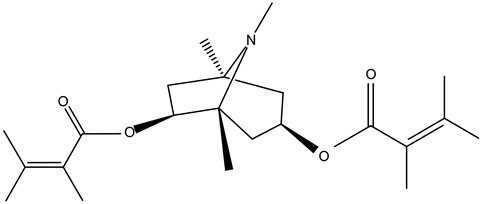 3,6-ditigloyloxytropane	
			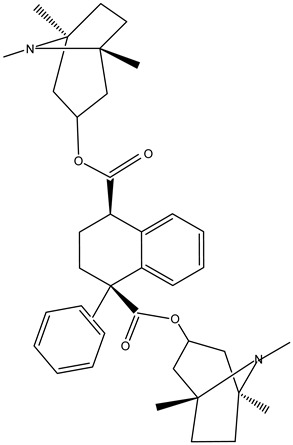 Beta-belladonnine	
			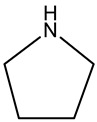 Pyrrolidine	
			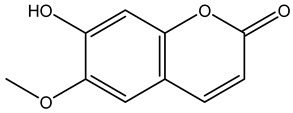 Scopoletin	
			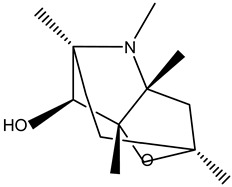 Scopoline	

**Table 4:** Antioxidant activities of *Mandragora autumnalis* (IC: Inhibitory concentration; TE: Trolox equivalent).

Extract	Plant Part	Dose	Methods	Observations	References
Flavonoid fraction	Ripe fruit	20–120 µg/mL	1,1-diphenyl-2-picrylhydrazyl (DPPH) assay	-Strong antioxidant activity -IC_50_ 5.37 ± 0.41 µg/mL	[13]
-Methanolic extract -Acetone extract	Flowers	N/A	1,1-diphenyl-2-picrylhydrazyl (DPPH) assay	-73.09 mg TE/g extract (strong antioxidant activity) -39.30 ± 0.10 mg TE/g extract (mild antioxidant activity)	[45]
-Methanolic extract -Acetone extract	Flowers	N/A	Phosphomolybdenum method	-mild antioxidant activity for both extracts -1.20 mmolTE/g extract -1.14 mmolTE/g extract	[45]
-Methanolic extract -Acetone extract	Flowers	N/A	Cupric ion reducing (CUPRAC) method	-113.24 mgTE/g extract (high antioxidant activity) -77.47 mgTE/g extract (mild antioxidant activity)	[45]
-Methanolic extract -Acetone extract	Flowers	N/A	Ferric reducing antioxidant power (FRAP) method	-90.88 mgTE/g extract (high antioxidant activity) -50.41 mgTE/g extract (mild antioxidant activity)	[45]
-Methanolic extract -Acetone extract	Flowers	N/A	Metal-chelating activity on ferrous ions	-high antioxidant activity for both extracts -15.94 mgEDTA/g extract -15.61 mgEDTA/g extract	[45]
-Methanolic extract -Acetone extract	Leaves	N/A	1,1-diphenyl-2-picrylhydrazyl (DPPH) assay	-mild antioxidant activity for both extracts -51.44 ± 0.2 9 mg TE/g -33.19 ± 0.13 mg TE/g	[45]
-Methanolic extract -Acetone extract	Leaves	N/A	Phosphomolybdenum method	-1.03 mmolTE/g extract (mild antioxidant activity) -1.98 mmolTE/g extract (strong antioxidant activity)	[45]
-Methanolic extract -Acetone extract	Leaves	N/A	Cupric ion reducing (CUPRAC) method	-Mild antioxidant activities for both extracts -77.98 mgTE/g extract -86.55 mgTE/g extract	[45]
-Methanolic extract -Acetone extract	Leaves	N/A	Ferric reducing antioxidant power (FRAP) method	-Mild antioxidant activities for both extracts -53.29 mgTE/g extract -53.53 mgTE/g extract	[45]
-Methanolic extract -Acetone Extract	Leaves	N/A	Metal-chelating activity on ferrous ions	-11.64 mgEDTA/g extract (mild antioxidant activity) -6.15 mgEDTA/g extract (low antioxidant activity)	[45]
Ethanolic extract	Leaves	1.56–200 µg/mL	1,1-diphenyl-2-picrylhydrazyl (DPPH) assay	-High antioxidant activity -IC_50_ 54.14 µg/mL	[29]
Aqueous fraction	Leaves	1.56–200 µg/mL	1,1-diphenyl-2-picrylhydrazyl (DPPH) assay	-High antioxidant activity -IC_50_ 23.67 µg/mL	[29]
n-hexane fraction	Leaves	1.56–200 µg/mL	1,1-diphenyl-2-picrylhydrazyl (DPPH) assay	-Mild antioxidant activity -IC_50_ 208.5 ± 3 µg/mL	[29]
Aqueous-Methanol fraction	Leaves	1.56–200 µg/mL	1,1-diphenyl-2-picrylhydrazyl (DPPH) assay	-Mild antioxidant activity -IC_50_ 165.9 ± 13 µg/mL	[29]
Aqueous extract	Roots	12.5–100 µg/mL	1,1-diphenyl-2-picrylhydrazyl (DPPH) assay	-Strong antioxidant activity -IC_50_ 47.16 ± 0.41 µg/mL	[46]
*M.autumnalis* synthesized silver nanoparticles	Roots	12.5–100 µg/mL	1,1-diphenyl-2-picrylhydrazyl (DPPH) assay	-Strong antioxidant activity -IC_50_ 51.81 ± 0.10 µg/mL	[46]

**Table 5:** The antimicrobial activities of *M. autumnalis*.

Extract	Dose	Experimental Model	Main Results	References
Antibacterial				
Fruit flavonoid fraction	Dose range: 0.5 to 500 µg/mL	-Method: Microdilution technique -Microorganisms: Gram-positive bacteria: *Staphylococcus aureus*, *Enterococcus faecium* and Methicillin-Resistant *Staphylococcus aureus* Gram negative bacteria: *Shigella sonnie*, *Pseudomonas aeruginosa*, *Klebsiella pneumoniae*, and *Escherichia coli*	-Good activity against all of the tested bacterial strains -More effective against the *K. pneumoniae* strain	[13]
Extract of the leaves (ethanol extract, n-hexane fraction, aqueous -methanol fraction, aqueous fraction)	Dose range: 0.03 to 50 mg/mL	-Method: Microdilution technique -Microorganisms: Gram positive bacteria: *Bacillus subtilis* Gram negative bacteria: *Pseudomonas aeruginosa* and *Escherichia coli*	-Good activity of the ethanol extract and n-hexane fraction against *B. subtilis* and *P. auriginosa*, with MIC values of 25 mg/mL -No activity of aq-methanol and aqueous fractions	[29]
Aqueous extract of the roots	100 µg/mL	-Method: Disk diffusion method -Microorganisms: gram positive bacteria: *Staphylococcus aureus*, *Bacillus subtilis* Gram negative bacteria: *Pseudomonas aeruginosa*, and *Escherichia coli*	-Good activity against all of the tested bacterial strains -More effective against the *Bacillus subtilis* strain	[46]
Aqueous extract of the synthesized silver nanoparticles of *M. autumnalis* roots	100 µg/mL	-Method: Disk diffusion method -Microorganisms: Gram positive bacteria: *Staphylococcus aureus*, *Bacillus subtilis* Gram negative bacteria: *Pseudomonas aeruginosa*, and *Escherichia coli*	-Good activity against all of the tested bacterial strains -More effective against the *Pseudomonas aeruginosa* strain	[46]
Antifungal				
Fruit flavonoid fraction	Dose range: 0.5 to 500 µg/mL	-Method: Mircodilution technique -Microorganisms: *Epidermatophyton floccosum* and *Candida albicans*	-Potent antifungal activity against *C. albicans*, with MIC values of 6.25 ± 0.48 µg/mL. -Weak activity against *E. floccosum* with a MIC value of 12.5 ± 0.88 µg/mL	[13]
Extract of the leaves (ethanol extract, n-hexane fraction, aqueous-methanol fraction, aqueous fraction)	Dose range: 0.03 to 50 mg/mL	-Method: Microdilution technique -Microorganisms: *Candida albicans*	-n-hexane fraction exhibited antifungal activity towards *C. albicans*, with an MIC value of 12.5 mg/mL. Aqueous/methanol and aqueous fractions showed no activity at 50 mg/mL	[29]

**Table 6:** The anticancer effects of *Mandragora autumnalis*.

The Solvent Used for Extraction	Plant Part	Dose	Experimental Model	Observation	References
Ethanol/ethyl acetate/water extract	Flowers and fruits	0.1–1000 µg/mL	Cell availability assay Cell lines: A549, HaCat cells	-Selective toxicity on A549 cells, with IC_50_ 369.5 ± 42.1 µg/mL -Less toxicity against HaCat cells, with IC_50_ > 1000 µg/mL	[61]
Ethanol/ethyl acetate/water extract	Whole plant	0.1–1000 µg/mL	Cell availability assay Cell lines: A549, HaCat cells	-Selective toxicity on A549 cells, with IC_50_ 201.9 ± 30.7 µg/mL -Less toxicity against HaCat cells, with IC_50_ 645.7 ± 51.4 µg/mL	[61]
Ethanol crude extract	Leaves	0.06–4 mg/mL	-MTT assay -Cell lines: MCF-7, MDA-MB-231, HCT-116, A549, VERO, EMT6/P	-Most effective against MCF-7 cells, with IC_50_ 0.10 ± 0.01 µg/mL -Low activity against VERO normal cell line, with IC_50_ > 4 µg/mL -Significant decrease in tumor size in vivo with (−35.99%) compared with the untreated control group (+107.02%)	[14]
n-hexane fraction	Leaves	0.06–4 mg/mL	-MTT assay -Cell lines: MCF-7, MDA-MB-231, HCT-116, A549, VERO, EMT6/P	-Most effective against MCF-7 cells with IC_50_ 0.48 ± 0.02 µg/mL -Low activity against VERO normal cell line, with IC_50_ > 4 µg/mL	[14]
Aqueous fraction	Leaves	0.06–4 mg/mL	-MTT assay -Cell lines: MCF-7, MDA-MB-231, HCT-116, A549, VERO, EMT6/P	-Low cytotoxic effect against all cancerous cell lines -low activity against VERO normal cell line, with IC_50_ > 4 µg/mL	[14]
Aqueous-methanol fraction	Leaves	0.06–4 mg/mL	-MTT assay -Cell lines: MCF-7, MDA-MB-231, HCT-116, A549, VERO, EMT6/P	-Low cytotoxic effect against all cancerous cell lines -low activity against VERO normal cell line, with IC_50_ > 4 µg/mL	[14]

**Table 7:** The anti-enzymatic potential of *M. autumnalis* extracts against several conditions.

Extract	Dose	Experimental Model	Observation	Reference
Antidiabetic				
Ethyl acetate fraction of the methanolic extract of *M. autumnalis* fruits	0–600 mg/mL	In vitro inhibition of α-glucosidase enzyme assay	-Dose-dependent increase in α-glucosidase inhibition -IC_50_ against this enzyme was 39.81 ± 0.74 µg/mL	[13]
Ethyl acetate fraction of the methanolic extract of *M. autumnalis* fruits	0–600 mg/mL	In vitro inhibition of α-amylase enzyme assay	-Dose-dependent increase in α-amylase inhibition -IC_50_ against this enzyme was 72.44 ± 0.89 µg/mL	[13]
Ethyl acetate fraction of the methanolic extract of *M. autumnalis* fruits	0–0.53 µg/mL	GLUT4 Translocation to the Plasma Membrane	-Dose-dependent increase in GLUT4 translocation was significantly noticed	[13]
Acetone extract from the leaves	N/A	-In vitro inhibition of α-amylase enzyme assay -In vitro inhibition of α-glucosidase enzyme assay	-Highest α-amylase inhibitory activity of 1.86 mmolACAE/g -No inhibitory activity of α-glucosidase	[45]
Acetone extract from the flowers	N/A	-In vitro inhibition of α-amylase enzyme assay -In vitro inhibition of α-glucosidase enzyme assay	-Mild α-amylase inhibitory activity of F-Ac of 1.27 mmolACAE/g -Mild inhibitory activity of α-glucosidase	[45]
The methanolic effect from the leaves	N/A	-In vitro inhibition of α-amylase enzyme assay -In vitro inhibition of α-glucosidase enzyme assay	-Mild α-amylase inhibitory activity of 0.51 mmolACAE/g -Mild inhibitory activity of α-glucosidase	[45]
Methanolic extract from the flowers	N/A	-In vitro inhibition of α-amylase enzyme assay -In vitro inhibition of α-glucosidase enzyme assay	-The least α-amylase inhibitory activity of 0.46 mmolACAE/g -Mild inhibitory activity of α-glucosidase	[45]
Anti-lipase				
Ethyl acetate fraction of the methanolic extract of *M. autumnalis* fruits	0–500 mg/mL	In vitro inhibition of lipase enzyme assay	-Dose-dependent increase in lipase inhibition -IC_50_ against this enzyme was 39.81 ± 1.23 µg/mL	[13]
Anticholinesterase and anti-tyrosinase				
Acetone extract from the leaves	N/A	-Cholinesterase (ChE) inhibitory activity using Ellman’s method to assess the ability to inhibit AChE and BCh -Tyrosinase inhibitory activity was measured using the modified dopachrome method with L-DOPA as substrate	-Most potent inhibitory activity on BChE -Highest tyrosinase activity, with 29.68 mgKAE/g extract	[45]
Acetone extract from the flowers	N/A	-Cholinesterase (ChE) inhibitory activity using Ellman’s method to assess the ability to inhibit AChE and BCh -Tyrosinase inhibitory activity was measured using the modified dopachrome method with L-DOPA as substrate	-Least potent inhibitory activity on BChE -Mild tyrosinase activity	[45]
The methanolic effect from the leaves	N/A	-Cholinesterase (ChE) inhibitory activity using Ellman’s method to assess the ability to inhibit AChE and BCh -Tyrosinase inhibitory activity was measured using the modified dopachrome method with L-DOPA as substrate	-Potent inhibitory activity on AChE and BChE -Mild tyrosinase activity	[45]
The methanolic extract from the flowers	N/A	-Cholinesterase (ChE) inhibitory activity using Ellman’s method to assess the ability to inhibit AChE and BCh -Tyrosinase inhibitory activity was measured using the modified dopachrome method with L-DOPA as substrate	-Most potent activity for AChE -No inhibitory activity against tyrosinase	[45]

## Data Availability

Not applicable.
